# Recurrent Endometrial Cancer: Local and Systemic Treatment Options

**DOI:** 10.3390/cancers13246275

**Published:** 2021-12-14

**Authors:** Heidi Rütten, Cornelia Verhoef, Willem Jan van Weelden, Anke Smits, Joëlle Dhanis, Nelleke Ottevanger, Johanna M. A. Pijnenborg

**Affiliations:** 1 Department of Radiation Oncology, Radboudumc, 6525 GA Nijmegen, The Netherlands; Lia.Verhoef@radboudumc.nl; 2 Department of Obstetrics & Gynaecology, Radboudumc, 6525 GA Nijmegen, The Netherlands; willemjan.vanweelden@radboudumc.nl (W.J.v.W.); Anke.Smits@radboudumc.nl (A.S.); Hanny.MA.Pijnenborg@radboudumc.nl (J.M.A.P.); 3 Faculty of Medical Sciences, Radboud University, Houtlaan 4, 6525 XZ Nijmegen, The Netherlands; j.dhanis@ru.student.nl; 4 Department of Medical Oncology, Radboudumc, 6525 GA Nijmegen, The Netherlands; Nelleke.Ottevanger@radboudumc.nl

**Keywords:** endometrial cancer, recurrence, treatment, surgery, radiotherapy, hormonal therapy, systemic treatment, oligometastases

## Abstract

**Simple Summary:**

In this review, we discuss the different treatment strategies in recurrent endometrial cancer. The incidence of endometrial cancer is rising. The available treatment options increase with the development of novel radiotherapy techniques and new systemic therapies. Dependent on the site of recurrence and previous therapy, the treatment of recurrent endometrial cancer can be curative or palliative. Newly emerging medical treatments, such as immunotherapy, might be of benefit in selected patients. Moreover, combinations of different treatments can lead to a better outcome. Recent insights on oligometastatic disease lead us to expect that ablative or radical local treatment for distant metastasis will be of benefit in selected patients. Due to the complexity of the cases, it is recommended to discuss individual cases in a multidisciplinary tumor board. Shared decision-making principles are recommended to maximize treatment personalization.

**Abstract:**

The treatment of recurrent endometrial cancer is a challenge. Because of earlier treatments and the site of locoregional recurrence, in the vaginal vault or pelvis, morbidity can be high. A total of about 4 to 20% of the patients with endometrial cancer develop a locoregional recurrence, mostly among patients with locally advanced disease. The treatment options are dependent on previous treatments and the site of recurrence. Local and locoregional recurrences can be treated curatively with surgery or (chemo)radiotherapy with acceptable toxicity and control rates. Distant recurrences can be treated with palliative systemic therapy, i.e., first-line chemotherapy or hormonal therapy. Based on the tumor characteristics and molecular profile, there can be a role for immunotherapy. The evidence on targeted therapy is limited, with no approved treatment in the current guidelines. In selected cases, there might be an indication for local treatment in oligometastatic disease. Because of the novel techniques in radiotherapy, disease control can often be achieved at limited toxicity. Further studies are warranted to analyze the survival outcome and toxicity of newer treatment strategies. Patient selection is very important in deciding which treatment is of most benefit, and better prediction models based on the patient- and tumor characteristics are necessary.

## 1. Introduction

Endometrial cancer (EC) is the most common gynecological cancer in the Western world. Its incidence is rising as risk factors for endometrial cancer are more and more prevalent [1,2]. The risk factors for developing endometrial cancer include prolonged unopposed estrogen exposure, advanced age, and obesity [3]. Most patients are diagnosed in their sixth or seventh decade, and, since postmenopausal blood loss is one of the first symptoms, most patients present with early-stage disease [4].

Endometrial cancer can be classified in different ways. Historically, two subtypes were recognized [5]. Type one tumors are the most common (70% of all tumors) and are predominantly well to moderately differentiated endometroid tumors with often high expression of the estrogen–progesterone receptor (ER/PR), carrying a good prognosis with surgery alone. Type two tumors are poorly differentiated endometroid carcinomas or more aggressive subtypes, such as clear cell or serous carcinomas. Type two tumors tend to be more advanced at diagnosis and have a poorer prognosis [6]. Nowadays, the classification according to the molecular profile is upcoming with tumor types such as *POLE*, MSI, and *P53*, where the first two predominantly have a favorable prognosis and the latter is considered to be an aggressive subtype [7,8].

**Figure 1:**
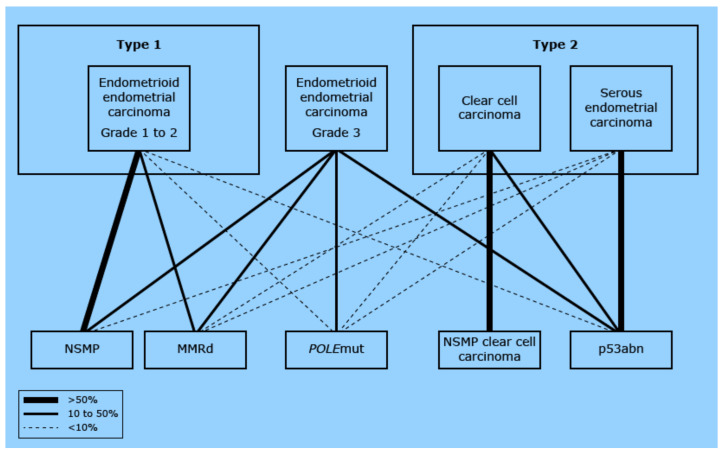
Relationship between the traditional histologic classification and the molecular classification. Each traditional histologic diagnosis is connected to the representing molecular subgroup. The thicker the connecting line, the stronger the relationship. The figure demonstrates that each molecular subgroup can be detected in each histologic subgroup. Yet, the NSMP is mainly reflected by grade 1 and 2 EEC (left in the figure), whereas the p53abn cancers are mainly reflected by patients with SC (right in the figure). EEC: endometrioid endometrial cancer; CCC: clear cell carcinoma; SC: serous cancer; NSMP: nonspecific molecular profile; MMRd: mismatch repair deficient; POLE: POLE ultramutated; p53abn: copy number high/TP53 mutated. (Modified from [9] UpToDate Endometrial cancer: Pathology and classification by Huvila J, MD, PhD, McAlpine JN, MD, FACOG, FRCPSC, available from: URL: https://www.uptodate.com/contents/endometrial-cancer-pathology-and-classification?source=history_widget) accessed 17 September 2021.

The relationship between the different histological subtypes and molecular profiles is shown in Figure 1. As illustrated, there is quite some overlap in the historically type two tumors and the more aggressive molecular subtypes, whereas the type one tumors tend to have a more favorable molecular profile.

The primary treatment consists of the surgical removal of the uterus and adnexa with or without lymph node dissection, or the sentinel node procedure. The rationale for nodal staging is based on the risk factors, i.e., tumor grade, deep myometrial invasion, or, with increasing evidence, molecular profile, but remains a subject of discussion [10,11].

The adjuvant treatment is based on the risk of locoregional recurrence or metastasis and can be locoregional radiotherapy, chemotherapy, or a combination of both. Patients with low-risk tumors can be treated with surgery alone, whereas patients with high-risk tumors are eligible for adjuvant chemotherapy and/or radiotherapy. There is no real international consensus about treating intermediate-risk tumors. The treatment strategies can vary between surgery only, adjuvant brachytherapy or pelvic radiotherapy, and/or chemotherapy [10,11].

Despite optimal surgical and adjuvant treatment, 7–15% of early stage (I-II) patients present with recurrent disease [12,13,14]. This can be locoregional recurrence, distant metastasis, or both. The risk of locoregional recurrence is low [12,15,16] and strongly related to the presence of risk factors, such as LVSI, tumor grade, or molecular profile [14]. About 50% of the patients with a recurrence have locoregional disease, 25% present with distant recurrence, and the remaining 25% have both [14]. Patients with advanced-stage disease at diagnosis or with a more aggressive subtype have a higher probability of both locoregional and distant recurrence [17]. Several studies showed a relapse-free survival of 60 to 70% after complete debulking and adjuvant (chemo)radiation in locally advanced disease [17,18]. Most recurrences occur within three years after the primary treatment, with a median 5-year survival of 55% after pelvic recurrence and 17% after metastatic disease [12,14]. 

The treatment options are dependent on the site of recurrence, tumor histology, i.e., biomarkers, previous treatments, and the patient’s performance status and preferences. The treatment strategies can include surgery, radiotherapy, hormonal treatment, systemic treatment, or a combination [19]. In this review, we will give an overview of the local and systemic treatment options for patients with recurrent endometrial cancer and intend to provide insight regarding the different treatment strategies for patients with an increasingly personalized approach in the future. 

## 2. Surgical Treatment for Locoregional Recurrence

### 2.1. Vaginal Vault Recurrence

For a vaginal recurrence in previously irradiated patients, surgical resection is considered to be the first-line treatment option, followed by adjuvant local radiotherapy (i.e., image-guided brachytherapy) if indicated [13,20,21].

Vaginal vault recurrences in non-irradiated patients are traditionally salvaged with radiation therapy [13]. However, the tumor size significantly influences the effect of radiotherapy and, therefore, surgical resection may be considered as a viable alternative. Wylie et al. showed that local control with radiotherapy was significantly worse for tumors >2 cm compared to smaller tumors (54% versus 80%) [22]. With the use of a combination of external beam radiotherapy (EBRT) and brachytherapy, also in larger tumors, a good locoregional control can be achieved [23]. Although Haldarson et al. showed comparable survival in patients with surgical resection and radiotherapy, in previously non-irradiated patients, the limited number of patients warrants further research [24].

### 2.2. Locoregional and Abdominal Recurrence

Historically, the role of surgery for recurrent endometrial cancer has been focused on exenterative surgery. Pelvic exenteration (anterior, posterior, or total) is performed with curative intent and has primarily been described in highly selected patients with an isolated pelvic adenocarcinoma recurrence after radiotherapy [25]. The complete resection of the tumor is reported to be feasible in the majority of patients, with the reported complete resection rates varying from 86% to 100% in a small case series [25,26,27]. However, the selection criteria are lacking, and the results are based on a small number of patients that have been selected retrospectively over long time periods. In addition, resection is accompanied by significant surgical-related morbidity and mortality with major complications in up to 80% of patients and mortality rates of 5% [25,27]. The five-year survival rates after pelvic exenteration are reported to be up to 56 to 70% when complete resection has been obtained (R0) compared to 20% or less in the presence of residual disease (R1-2) [25,26,27].

Over the past years, the role of surgical cytoreduction (i.e., excision of all visible disease) for recurrent endometrial cancer has been gaining increasing interest as a surgical alternative with less morbidity compared to exenteration. Retrospective studies have demonstrated the feasibility and additional value of cytoreductive surgery [28,29,30,31,32]. Patient selection was based upon a local multi-disciplinary team review and included endometroid and non-endometroid tumors. Most studies included both locoregional (pelvic and nodal) and intra-abdominal recurrences. After cytoreduction, 40–60% of the patients were treated with systemic therapy, radiotherapy, or both [21,33,34]. The removal of all visible disease was achieved in the majority of the patients, varying from 56% to 71% [21,29,33]. The factors associated with complete cytoreduction were: solitary recurrence, tumor size (<6 cm), and performance status [30,32,33]. Advanced age and the presence of peritonitis carcinomatosis negatively impacted the achievement of complete cytoreduction and survival [32,34]. The surgical morbidity rates varied from 9% to 21%, mainly grade one and two complications, and no perioperative deaths were reported [28,29,30,32].

Complete resection was significantly associated with improved overall survival [21,28,31,32]. A recent multi-institutional study of 230 patients reported a significantly improved 5-year survival of 66% in patients with no residual disease and negative resection margins compared to 37% in patients with macroscopic residual disease. The site of recurrence did not impact the survival outcomes [31]. In a large meta-analysis by Barlin et al., the role of cytoreductive surgery for both advanced (n = 515) and recurrent (n = 157) endometrial cancer patients was evaluated. Complete cytoreduction was associated with a superior overall survival outcome, with each 10% increase in proportion with the patients undergoing complete cytoreduction showing a 9-month increase in survival. In the cases with residual disease of 2 cm or more, the survival benefit was lost, supporting proper selection and the aim to achieve complete debulking [35]. 

Whether neoadjuvant chemotherapy in a recurrent setting might be a valuable treatment option prior to debulking has not been studied so far. Based on the data in the primary setting, neoadjuvant chemotherapy in advanced-stage disease resulted in 80% complete debulking [36] and, as such, could be considered in individual patients with a good performance status.

### 2.3. Solitary Distant Metastasis

There are few reports on the surgical management of isolated distant recurrences. Tangjitgamol et al. reviewed the role of the surgical resection of solitary pulmonary, hepatic, and cerebral metastasis and deemed it feasible for individualized cases. The successful resection of splenic metastasis has also been reported [37]. The favorable prognostic factors for a prolonged survival were good performance status, long disease-free interval, and clear margins [38]. However, the studies are limited and comprise mainly case reports, warranting further research before evidence-based guidelines can be drafted.

## 3. Radiotherapy

### 3.1. Vaginal Vault Recurrence

In the PORTEC-1 trial, the locoregional recurrence was 14% in previously unirradiated patients with 11% vaginal recurrence and 3% pelvic recurrence. In previously irradiated patients, 4% locoregional relapse occurred, of which 2% was pelvic recurrence [15]. This is in line with other published recurrence rates, such as Francis et al., who found 7% overall recurrences with 4% only locoregional recurrences in 2691 patients with stage I-II endometrial cancer [16].

The treatment of a vaginal recurrence usually requires a combination of external beam radiotherapy (EBRT) with elective nodal irradiation and brachytherapy boost. In radiation-naïve patients, this is often the treatment of choice. Local control is obtained in 60–80% with acceptable toxicity, mostly gastrointestinal and urogenital toxicity [13]. The current consensus is that a cumulative dose of 80 Gy in the target volume should be reached in order to achieve >90% local control [23,39]. MRI-guided brachytherapy techniques are required to safely reach such doses with minimizing the dose to the organs at risk, and, in many cases, laparoscopic guidance during applicator placement is needed to reach adequate target coverage and avoid bowel perforation [40]. Isolated vaginal vault recurrences after previous postoperative vaginal vault brachytherapy can be treated likewise, without dose-adjustment. 

### 3.2. Pelvic Recurrence

Local control is worse for patients with a pelvic recurrence as opposed to a vaginal recurrence with reported 5-year local control rates between 30 and 60% [41,42]. In addition, the overall survival is better for patients with a vaginal versus pelvic nodal recurrence, respectively, 73% and 8–14% [41]. 

Patients with a pelvic recurrence can be treated with EBRT with a boost to the macroscopic lesions. In these cases, a combination of radiation and chemotherapy or surgery might be beneficial as well, and this needs to be individualized [16,41]. 

In previously irradiated patients, the incidence of locoregional recurrence is lower, but treatment of a recurrence is more challenging. The achievable dose will be considerably lower in such cases and mainly depends on the tolerance of the nearby organs at risk, particularly the bowel [23]. Re-irradiation has long been controversial because of the high incidence of toxicity (fistula, bowel/bladder toxicity) [43]. However, with the improvement of techniques integrating imaging during radiotherapy, this might be overcome in selected cases. Nowadays, re-irradiation with stereotactic irradiation on a conventional linac or MRLinac (MRL) is feasible and with acceptable toxicity [44,45].

### 3.3. Oligometastases

Recent developments in stereotactic radiotherapy have facilitated safe irradiation of several malignant lesions to an ablative dose. Up to five lesions are considered ‘oligometastatic disease’, and the SABR-COMET study has demonstrated a prolonged disease free- (DFS) and overall survival (OS) after stereotactic radiotherapy compared to standard palliative radiotherapy in patients with a variety of primary tumors [46]. Scarce retrospective data appear to confirm these results for gynecological cancer patients [47]. 

### 3.4. MR Linac (MRL)

Stereotactic body radiotherapy (SBRT) is a method of EBRT that accurately delivers a high dose of irradiation in one or a few treatment fractions to an extracranial target [48]. To safely deliver these high doses, image guidance is necessary. In the case of lung or bone metastases, cone-beam CTs (CBCT) will be sufficient for adequate image guidance. Hence, most modern LINACS equipped with CBCT can deliver SBRT for these disease sites. CBCTs are not sufficient to properly visualize malignant lesions in the abdomen and viscera, such as nodal and liver metastases.

MR guided stereotactic radiotherapy, as is performed on an MRL, is now emerging as a treatment modality for SBRT in body sites that were recently too difficult to visualize on CT. The MR LINACS are integrated imaging and radiation systems, which enables visualization of the target immediately before, during, and after irradiation. The irradiation of targets very close to sensitive organs, such as the small bowel, employment of smaller margins, and adjustment of the treatment to the anatomy on a daily basis, are now possible. Abdominal organs tend to move, dependent on bladder or bowel filling, and daily plan adjustment allows the radiation oncologist to give a high dose while still sparing the organs at risk. This feature makes MR LINAC-based SBRT very appropriate for re-irradiation of small recurrences in previously irradiated areas, such as nodal recurrences in the abdomen. Currently, experience is still limited, but centers worldwide are gathering evidence about the effectiveness of MR-based SBRT [49].

**Figure 2:**
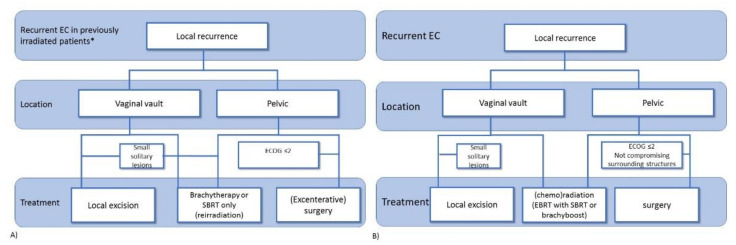
Flow chart for possible treatment decisions in patients with recurrent endometrial cancer after initial surgical treatment with (**A**) or without (**B**) previous adjuvant locoregional radiotherapy. EBRT: external beam radiotherapy; SBRT: stereotactic body radiotherapy. * excluding adjuvant brachytherapy only.

In summary, the treatment of locoregional recurrence can be either with (chemo)radiotherapy or by surgical resection and is dependent on whether or not previous pelvic radiotherapy has been applied. In Figure 2, possible flowcharts for recurrent endometrial cancer in previously irradiated patients (Figure 2A) and in patients who did not undergo previous pelvic radiotherapy (Figure 2B) are shown. The balance between potential toxicity and benefit should be discussed with each individual patient. In the case of oligometastatic disease, local treatment might be of benefit. 

## 4. Systemic Treatment

### 4.1. Chemotherapy

In the chemotherapy trials for recurrent endometrial cancer, almost all the randomized studies also included patients with locoregionally advanced endometrial cancers, which might result in more favorable results compared to those patients with metastatic disease. 

The publication of randomized trials concerning systemic chemotherapy for metastatic endometrial cancer started in the late previous century with doxorubicin(A), later combined with cisplatin (P) and paclitaxel (T) [50,51,52,53]. TAP was, for a long period, the most effective evidence-based therapy with a significantly higher response rate of 57% versus 34% for AP (P < 0.01), and improved PFS (median, 8.3 v 5.3 months; P < 0.01), and OS (median, 15.3 v 12.3 months; P = 0.037). However, toxicity, and especially neurological toxicity, as well as cardiac toxicity, were a major concern in this elderly population, and many centers started to use carboplatin and paclitaxel instead, with similar results. In 2020, the long-awaited randomized non-inferiority study GOG0209 comparing carboplatin and paclitaxel with paclitaxel, doxorubicin, and cisplatin confirmed that carboplatin and paclitaxel is not inferior to TAP [54].

Only a few randomized phase III and phase II chemotherapy trials have been published since, investigating schedules for a second recurrence, albeit without startling effects. McMeekin et al., published an early stopped, phase III trial of ixabepilone versus either paclitaxel or doxorubicin for second-line treatment [55]. Docetaxel combined with platinum did not seem superior to paclitaxel [56,57], and neither was vinorelbine combined with cisplatin [58], nor topotecan [59], dactinomycin [60], pegylated doxorubicin [61,62], oxaliplatin [63], pemetrexed [64], trabectedin [65], or gemcitabine [66]. A weekly schedule with carboplatin and paclitaxel according to a phase II trial seems to have one of the more favorable results for patients previously treated with chemotherapy with a response rate of 39% and a median PFS of 8 months and an OS of 9 months at the cost of increased myelodepression and neuropathy [67].

### 4.2. Immunotherapy

Immunotherapy with checkpoint inhibitors, both PD1 and PDL1 inhibitors, is, nowadays, the most promising therapy for endometrial cancer. Two drugs are currently approved by the EMA and/or FDA; pembrolizumab and dostarlimab. Dostarlimab (Jemperli) was granted accelerated approval both by the FDA and EMA for the treatment of patients with recurrent or advanced deficient mismatch repair (dMMR) endometrial cancer that has progressed or following prior treatment with platinum-containing chemotherapy [68]. In patients with previously treated metastatic endometrial cancer irrespective of MSI/MMR status, a response rate of 64% in MSI-H/MMRD and 36% for MSS/pMMR was seen with the combination of pembrolizumab and lenvatinib [69]. Based on this study, pembrolizumab with lenvatinib was also approved in an accelerated manner by the FDA for patients with previously treated metastatic endometrial cancer whose tumors were not MSI-H/dMMR. This study was followed by KEYNOTE-775/Study 309, a randomized phase III trial for endometrial cancer patients with tumors that are not deficient mismatch repair or MSI high and who have recurrent disease following prior systemic therapy. For this combination of lenvatinib and pembrolizumab, the median OS improved from 12 months to 17.4 months, with an HR 0.68 (95% CI 0.56–0.84) (Makker (abstract SGO 2021). The marketing authorization application is currently under review by the EMA. The results from other Checkpoint inhibitor phase III trials with, for example, durvalumab with olaparib (NCT 04269200) and atezolizumab with carboplatin and paclitaxel (NCT 03603184), will become available in the future years.

### 4.3. Targeted Therapy

A wide range of targeted therapies have been explored for metastatic endometrial cancers. In the early years when these drugs became available, they were used in unselected patients; more recently, due to increased availability of genome sequencing, more studies are focused on specific genetic alterations, such as mutations and copy number changes in the tumor. No randomized phase III trials have been published for targeted therapies in this patient population. Neither has any targeted drug been approved by the FDA or EMA at this moment. A large number of randomized phase II and phase III trials have been published on angiogenesis inhibitors, such as bevacizumab [70,71,72], brivanib [73], nintedanib [74], sunitinib [75], cediranib [76], trebananib [77] and lenvatinib [78], thalidomide [79], and aflibercept [80]. Another group of drugs of interest in endometrial cancers are the mTOR inhibitors: temsirolimus [71,81,82,83,84], ridaforolimus [85,86,87], and everolimus [88,89] again without success for registration. The newer drugs, such as PIK3CA inhibitors and AKT inhibitors, have been tested in few phase II trials, with only one looking at a PIK3CA mutation upfront [90] In addition, several EGFR inhibitors were used in phase II trials, such as gefitinib [91] and erlotinib [92]. The MEK inhibitor selumetinib also did not pass to a phase III trial [93]. Selinexor, an exportin 1 inhibitor, showed interesting results in endometrial cancer, and this is one of the rare targeted drugs proceeding to a phase III trial. It is currently being tested as maintenance after a response to carboplatin paclitaxel (NCT03555422). 

## 5. Hormonal Treatment

Hormonal therapy for endometrial cancer has been used since the 1950s after it became clear that progesterone could induce the regression of endometrial hyperplasia and EC [94]. In the first publication by Kelley and Baker in 1961, a response rate of 29% to progestin therapy was reported among 21 recurrent ECs. This led to widespread application of progestin therapy in clinical practice. Initially, several investigations in patients with advanced-stage and recurrent EC confirmed the original reported response rates, with some studies observing a response rate as high as 56% of patients [95,96]. However, recent studies with a more modern trial design and more stringent endpoints reported a lower response rate ranging from 11% to 24%, although patients that did respond often had long progression-free intervals [97,98,99]. Newer hormonal drugs, such as tamoxifen and aromatase inhibitors, have shown lower response rates than progestins and are, therefore, regarded as second-line hormonal therapies [100,101].

The response rates are significantly higher in ER/PR positive EC [102,103]. Nevertheless, a recent meta-analysis showed that ER/PR status was integrated in only 70 out of 1837 included cases, indicating the limited available research on ER/PR status and response to hormonal therapy in EC. Van Weelden et al. demonstrated that the classification of ER/PR expression into three risk groups (0–10% (high), 20–80% (intermediate), and 90–100% (low)) resulted in better prognostication in EC, suggesting tissue specific cut-off [104]. Multiple studies have shown that, during cancer progression, the loss of ER/PR occurs in at least 20% of metastasis from ER/PR positive primary tumors; this underlines the relevance of reassessing ER/PR prior to the start of the treatment of recurrent EC [105]. 

Yet, the presence of ER/PR is not inherently reflecting active intracellular ER signaling and hormone driven tumor growth. Therefore, the ER pathway activity testing that indicates an activated ER signaling might improve the prediction of the response to hormonal therapy in EC. In EC, ER pathway activity scores (ERPAS) were demonstrated to better predict the prognosis compared to ER expression [106]. In a recently published paper, pretreatment biopsies of patients with recurrent (n = 51) and advanced (n = 30) endometrial carcinoma were analyzed for ER/PR expression and integrated the ERPAS analysis. Interestingly, all the responders, i.e., complete- and partial response (CR, PR), had ER/PR expression >50%. Among progestin users, the response rate (RR) was 37.7% for ER > 50%, 56.8% for PR > 50%, and 62.1% if activated ERPAS [107]. In a multivariable regression analysis, including tumor grade, histology, ER/PR, and ERPAS, the ERPAS >15 was the sole marker that remained significantly associated with PFS (HR 4.525, 95%-CI 1.85–11.07, *p* = 0.001). A multivariable regression analysis without ERPAS showed that PR expression was the only variable with significant association with PFS (HR 2.964, 95%-CI 1.58–5.58, *p* = 0.001). In those who responded to hormonal therapy, 34.3% of the patients with PR >50% had not progressed after 2 years.

**Figure 3:**
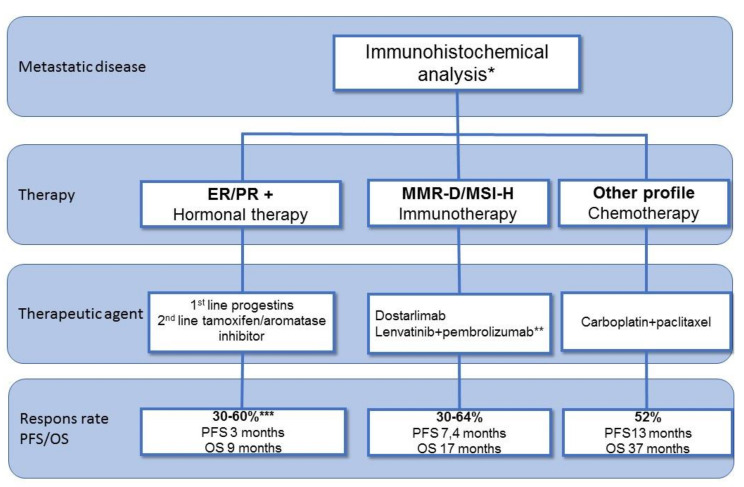
Overview of systemic treatment options in recurrent endometrial cancer. ER: estrogen receptor; PR: progesterone receptor; MMR-D: mismatch repair deficient; MSI: microsatellite instability; MSS: microsatellite stable; pMMR: proficient mismatch repair; PFS: progression-free survival; OS: overall survival. * Preferably histology on recurrent tumor ** also approved for pMMR/MSS *** Dependent on level of expression.

In conclusion, patients with systemic recurrent endometrial cancer can be treated with hormonal therapy, immunotherapy, or chemotherapy. The evidence on targeted therapy is limited. Figure 3 summarizes the systemic treatment options with response rates and data on progression-free survival and overall survival. 

## 6. Conclusions and Future Perspectives

The treatment of recurrent endometrial cancer is a therapeutic challenge, especially in the previously irradiated patient or in the patient with oligometastatic disease. In the last decade, the improved selection of patients with recurrent endometrial cancer resulted in an improved 5-year survival rate from 25% up to 75%. The treatment modalities can be either local (surgery and radiotherapy) or systemic (chemotherapy, targeted therapy, hormonal therapy, or immunotherapy). In the case of systemic therapy, evidence is available for chemotherapy, immunotherapy, and hormonal therapy. In the case of targeted therapy, so far, no phase III trials are available, hampering specific recommendations. It is expected that molecular profiling in endometrial cancer will be directive not only in the adjuvant setting but also in patients with recurrent disease [108]. Furthermore, combinations of local and systemic treatment might benefit selected patients. Trials with combinations of radiotherapy and immunotherapy or combinations of different systemic treatments are ongoing, and, hopefully, more evidence will become available in the following years. To gain better evidence regarding the different treatment strategies, further studies are warranted. Furthermore, to better select patients, research in the field of predictive biomarkers and the prospective analysis of outcome in large databases is necessary.

Close collaboration between the radiation oncologist, medical oncologist, pathologist, radiologist, and gynecologic surgeon is essential to obtain the best possible outcome for these complex patients, and discussing these patients in a multidisciplinary tumor board with experience in treating recurrent endometrial cancer is mandatory.
